# Epigenetics—Shedding Light on the Path Ahead for Material Sciences

**DOI:** 10.3390/diseases7020043

**Published:** 2019-06-14

**Authors:** Anne Krüger-Genge, Olivia Mauger, Joachim Storsberg, Christian Schmidt

**Affiliations:** Department of Biomaterials and Healthcare, Fraunhofer-Institute for Applied Polymer Research (IAP), Division of Life Science and Bioprocesses, 14476 Potsdam-Golm, Germany; Anne.Krueger-Genge@iap.fraunhofer.de (A.K.-G.); Olivia.Mauger@iap.fraunhofer.de (O.M.); Joachim.Storsberg@iap.fraunhofer.de (J.S.)

**Keywords:** epigenetics, medical devices, material sciences

## Abstract

The harmonious regulation of bodily function is a necessity for healthy individuals. Looking from the viewpoint of material sciences, one can only marvel at the cellular factories, their renewal, and the overall control of messaging and control of responses. As aging progresses and/or pathologies arise, clinicians may be forced to look for replacement of organs/tissues with medical devices. Since all devices are tailored, a detailed understanding of developmental processes, including aberrant processes leading to pathologies, is crucial to provide clinicians with a suitable device. Although research in the field of epigenetics has produced effective therapeutics and diagnostic markers, our currently fragmented understanding of epigenetic processes as they relate to material development is inherently limited, with logical implications for the success of medical procedures. Here, we illustrate how material sciences for clinical applications, critically depend on all aspects of biomedical sciences, including the field of epigenetics.

## 1. Discussion

Reducing the posed question of providing personalized devices to patients in need to a more generalized problem of development forces one to concede that the literature does not provide a simple and straightforward answer. In more precise wording, generalizations across species are not encouraged, nor is there a consensus on an understanding of apparently dualistic phenomena, termed ‘repeatability of development’ and ‘inverse of developmental *noise*’ [1]. Adding complexity to an already intricate problem involves the notion whether "the whole genetic system … as a set of potentialities for developmental reactions and as a set of heritable units" [2] can be likened, at an abstract level, to the concept of tonal notation with an implied substantial range of outcomes. At first glance, this may look like an arcane question for a philosopher. If, however, this problem is examined and considered with an independent mind [3], answers to this question are certainly doing the public some good [4], benefitting clinical research and material sciences alike [5].

A case in point could be mounted by examining the preference of human traits during human development in an environment with limited resources. In such a case, advantageous traits such as energy storage [6] now lead to a disadvantage in populations in terms of relative ease of satisfying basic needs [7], although a generalized view on a wider range of developmental pressures remains to be brought to light [8].

Particularly pressing is the development of personalized and high-performing dental implants. Diverse methodologies and study groups hinder present-day extraction of structured knowledge from individual studies to clarify the influence of epigenetic phenomena on the performance of dental implants [9,10,11]. Similarly, corneal wound healing [12] and senile cataract [13,14,15] are areas in which material science is awaiting the infusion of new research findings to provide sufficient clues as to what the material should do in order to help the patient and the clinician. Looking at earlier precedence [16], one may realize that the problem of implants as foreign objects is still a problem to be addressed.

To illustrate the point from another perspective, material sciences, in this case developing formulations for anti-cancer-medication, relies upon the principle of trial and error. However, one publication suggests an alternative path [17]. The authors are not going so far as to suggest they found the holy grail, but getting a sense of how to streamline the pipeline would probably be seen as advantageous by many. Furthermore, one need not assume that this feat may be accomplished by a singular effort or by a single sub-discipline [18].

To tackle the question from a third angle, we turn our attention to cells and tissues. It is apparent that our fragmented understanding of how cells sense their environment, such as through biophysical and dissolved cues, as individual classes of stimuli and their interplay with other signaling pathways, can best be illustrated by a surgeon’s choice of procedures to aid wound healing in hernia-type lesions on the one hand and an apparent paucity of formal studies to illuminate the phenomena involved on the other [19]. Similarly, a relatively confined area, termed stem cell niche, presents cells with a wealth of stimuli that are not completely understood with regards to input–output schemes and fail-safe mechanisms [19,20].

## 2. Conclusions

Where do we go from here? Do we throw up our hands and leave this topic alone? Emerging evidence connecting epigenetic phenomena with clinical needs, far from being adequately addressed, leaves no alternative but getting to the bottom of all this. This may provide material scientists from all corners with the intellectual foundation to help clinicians as they are confronted with manifestations that can, at this point, neither be managed, nor cured. “*I kid you not*” [4], epigenetics may very well be the candle in reach to shed light on the path ahead.
